# On the question of proportionality of the count of observed Scrapie cases and the size of holding

**DOI:** 10.1186/1746-6148-5-17

**Published:** 2009-05-06

**Authors:** Dankmar Böhning, Victor J Del Rio Vilas

**Affiliations:** 1 Quantitative Biology and Applied Statistics, School of Biological Sciences, University of Reading, Reading, RG6 6FN, UK; 2 Veterinary Laboratories Agency (VLA), New Haw, Weybridge, Surrey KT15 3NB UK; 3 Department for Environment, Food and Rural Affairs (Defra), Nobel House, 17 Smith Square, London, SW1P 3JR, UK

## Abstract

**Background:**

The present paper investigates the question of a suitable basic model for the number of scrapie cases in a holding and applications of this knowledge to the estimation of scrapie-affected holding population sizes and adequacy of control measures within holding. Is the number of scrapie cases proportional to the size of the holding in which case it should be incorporated into the parameter of the error distribution for the scrapie counts? Or, is there a different – potentially more complex – relationship between case count and holding size in which case the information about the size of the holding should be better incorporated as a covariate in the modeling?

**Methods:**

We show that this question can be appropriately addressed via a simple zero-truncated Poisson model in which the hypothesis of proportionality enters as a special offset-model. Model comparisons can be achieved by means of likelihood ratio testing. The procedure is illustrated by means of surveillance data on classical scrapie in Great Britain. Furthermore, the model with the best fit is used to estimate the size of the scrapie-affected holding population in Great Britain by means of two capture-recapture estimators: the Poisson estimator and the generalized Zelterman estimator.

**Results:**

No evidence could be found for the hypothesis of proportionality. In fact, there is some evidence that this relationship follows a curved line which increases for small holdings up to a maximum after which it declines again. Furthermore, it is pointed out how crucial the correct model choice is when applied to capture-recapture estimation on the basis of zero-truncated Poisson models as well as on the basis of the generalized Zelterman estimator. Estimators based on the proportionality model return very different and unreasonable estimates for the population sizes.

**Conclusion:**

Our results stress the importance of an adequate modelling approach to the association between holding size and the number of cases of classical scrapie within holding. Reporting artefacts and speculative biological effects are hypothesized as the underlying causes of the observed curved relationship. The lack of adjustment for these artefacts might well render ineffective the current strategies for the control of the disease.

## Background

Surveillance efforts must adjust to the levels of occurrence of disease especially in the face of multiple threats and finite resources. There is a need to consider, among other parameters that would inform the level of priority allocated to a given disease, its prevalence and, ideally, that adjusted for any source of under-ascertainment. We define ascertainment as the definitive and complete determination of individuals with a particular trait of interest, scrapie in our case. Readily available methods for the estimation of the under-ascertainment-adjusted holding prevalence and within holding prevalence are required when planning surveillance strategies and control measures in animal health settings.

The occurrence of classical scrapie, a fatal, neurological disease of small ruminants, appears to be decreasing in Great Britain, both in the number of holdings affected and the number of sheep infected within holding [1]. Such a decreasing trend will undoubtedly lead to the reconsideration by policy makers of the overall efforts dedicated to the surveillance of the disease.

The importance of the size of the holding in the occurrence of classical scrapie is well described in the literature [2-6]. The results from these studies have been used to parameterize mathematical models describing scrapie transmission between sheep and between flocks [7]. In this particular case, a positive linear relationship was assumed between the occurrence of the disease within holding and the size of the holding. Note that some of the studies above indicated some form of non proportionality between holding size and the occurrence of scrapie [5,6]. There is a need to describe this relationship to direct disease control measures in Great Britain. Sampling and TSE-testing of scrapie-affected flocks is one of the measures in place after the introduction of the Compulsory Scrapie Flocks Scheme (CSFS) in Great Britain in 2004 [8]. The calculation of the sample size is based on the number of adult sheep within the holding and increases proportional to the size of the holding (under the assumption of a perfect test). A non-linear relationship between the occurrence of the disease and the size of the flock might render these sample calculations ineffective for larger holdings at the right end of the size distribution. Furthermore, there is a need to inform this relationship not only when dealing with reported clinical disease, potentially affected by reporting artefacts. The count of scrapie cases arising from the TSE-testing within holding under the CSFS provides the data for the assessment of this relationship free from potential reporting biases and merits study.

Previous studies have applied capture-recapture (CRC) techniques to obtain adjusted estimates of the prevalence of scrapie-affected holdings in Great Britain [9]. Recently, two approaches [10,11] were pursued for the incorporation of observed holding-specific variability in their CRC models: via an extension of Zelterman's (1988) estimator either as a covariate in a logistic model [10] or as a proportional term [11]. The form of this relationship influences the estimates of the scrapie-affected holding population and requires further analyses.

Hence, our objective in this paper is three-fold:

i) To model the relationship between holding size and the occurrence of disease,

ii) To study the effects of this relationship in capture-recapture estimates of the size of the scrapie-affected holding population and

iii) To discuss the consequences for within-holding sampling schemes for the detection of the disease.

## Methods

### Materials

Several sets of analysis were conducted and for each we used different datasets. The first analysis used year-specific disease data from the Scrapie Notifications Database (SND) (see [6] for more details). The SND collects all the suspect clinical cases of scrapie reported by farmers to the veterinary authorities. More specifically, we restricted our analyses to the number of confirmed clinical cases for the years 2002, 2003 and 2004. This provided a snapshot of the relationship between the holding characteristic of interest, its size, and the presence of clinical disease. The year-specific datasets were also used to estimate the under-ascertainment-adjusted population of scrapie-affected holdings, with clinical disease, in Great Britain per year. Note that as a by product of the application of CRC models to the SND dataset, the surveillance's sensitivity, for the detection of scrapie-affected holdings with clinical disease, was estimated at around 40–50% [10].

A second set of analysis used the list of holdings sampled and TSE-tested within the CSFS during 2005 and 2006. More specifically, animals tested and confirmed from the initial cull (IC) route (see [8] for more details). In theory, this route deals only with healthy animals, randomly selected from the eligible cull population within holdings, so if any case arises it is likely to be pre-clinical. This set of analysis would inform the relationship between the number of pre-clinical cases and the size of the holding, free from reporting artefacts potentially affecting the SND results. We extended the analyses on this dataset to estimate the overall population size of scrapie-affected holdings (in the remainder of the paper simply referred to as the population size estimate). The three regular surveillance sources for scrapie in Great Britain, the SND and the fallen stock survey targeting clinical disease and the abattoir survey targeting infection (note that the SND is an exhaustive list and the two surveys are sample-based sources), feed cases into the CSFS. As a result, any population size estimate based on the CSFS would be comprehensive and represent the overall burden of the disease, regardless of its manifestation (i.e. clinical disease or infection). The authors showed the limitations of population size estimates based on CSFS data [12]. We have conducted these analyses for illustrative purposes to show the effect of an inadequate parameterisation on the model's results.

Note that for the estimation of the relationship between counts of cases and holding size for the CSFS dataset, index cases, those detected through the regular surveillance activities (for further details see [13,14]) that triggered the incorporation of the holdings in the CSFS, were initially included in the analyses. Due to the weight of the holdings detected through the SND in the CSFS dataset [12] we would expect similar results to those obtained from modelling the SND data alone. In a further analysis, we removed the index cases to reduce the impact of potential reporting artefacts. We extended this approach to study the relationship *between *the number of tested animals within a holding in the CSFS *and *the count of cases. Here we would expect to see a linear relationship to occur in the sense that the more animals we test the more we detect (assuming a constant within-holding prevalence across categories of holding size). A non-linear relationship between case count and number tested might reveal a deviation from the assumption of constant within-holding prevalence across holdings of different sizes.

For each year and holding, our unit of analysis, we obtained the holding size from the Census data [15].

### Statistical Methods

#### Relationship between holding size and number of cases

Consider the following setting. A count of scrapie cases *Y*_*i*_ is observed in holding *i *with size *n*_*i*_. The question is if *E*(*Y*_*i*_) is proportional to *n*_*i*_. If it were the prevalence would be determined by a constant ratio *E*(*Y*_*i*_)/*n*_*i *_= *μ*, say for all holdings. This can be written alternatively as log *E*(*Y*_*i*_) = log *μ *+ log *n*_*i *_for all holdings *i *= 1,..., *n*. This simple model can be further written as

(2.1)

with *α *= log *μ*, *x*_*i *_= log *n*_*i*_ and *β*_1 _= 1. Equation (2.1) with *β*_1 _= 1 is called an *offset model *since the coefficient is known and fixed to be *β*_1 _= 1. We call this **Model 1**. If we let *β*_1 _to vary freely, then (2.1) is the conventional log-linear model with one covariate *x*_*i*_, the log-size of the holding. The latter we call **Model 2**. Note that Model 1 is a special case of Model 2, thus it is *nested *within Model 2. It is also appropriate to see if there is any *curvature *in the model. Hence we consider

(2.2)

which we call **Model 3**. Note that Model 2 is nested within Model 3.

The parameters need to be estimated and this is done by means of maximum likelihood. An observed scrapie-affected holding is defined by having a non-zero confirmed count of scrapie cases within the holding. Hence zero-counts of scrapie affected holdings cannot occur. Consequently, a zero-truncated Poisson likelihood is the basis of the inference and since all models are nested, model comparisons can be achieved using the likelihood ratio test (LRT). Log-likelihoods, the Akaike Information Criterion (AIC) and the Bayesian Information Criterion (BIC) were computed for model selection. These models were applied to the SND and CSFS datasets. The latter with the index cases included.

#### Modelling for CSFS data with zero counts

For our next analyses we used the TSE-test results from the list of holdings in the CSFS in 2005 and 2006. Index cases were not incorporated into our models. This allowed us to focus on the count of cases arising from the unbiased TSE-testing within CSFS holdings. Also, holdings with 0 tested animals in the IC route had to be removed from the data set. There are now zero counts in the data set (those holdings with no detected scrapie after the TSE-testing) so that the conventional Poisson regression model could be considered. However, since there appear to be more zero-counts now than we would expect under the conventional Poisson model we consider also a *zero-inflated Poisson regression model *(ZIP) which is provided by

(2.3a)

if *y *= 0 and

(2.3b)

if y>0. Here, *Po*(*y*, *λ*) = *exp*(-*λ*)*λ*^*y*^/*y*! is the Poisson density and *λ *is the mean of the compartment representing the Poisson distribution. We point out that the ZIP-model is particularly suitable for modelling spikes at zero. Modelling proceeds then by fitting the additional parameter *p *and potential models for the mean *λ *as a function of size *n*_*i *_in the holding *i*:



with *α *= log *μ*, *x*_*i *_= log *n*_*i *_and *β*_1 _= 1. This is the offset-model if the slope parameter is fixed to *1*. More general models are possible by allowing the slope parameter to be arbitrary or adding curvature terms.

#### 2.2.3 Adjusting estimates of the size of the population of scrapie-affected holdings

Based on our results from the previous section, we can develop a capture-recapture estimator for the size of the scrapie-affected holding population. To demonstrate consider **Model 2 **log *E*(*Y*_*i*_) = *α *+ *β*_1_*x*_*i *_where estimates  and  for the parameters are found by maximum likelihood. Consider for each holding the *linear predictor * and construct the generalized Horvitz-Thompson estimator as suggested in [16]

(2.4)

where  is the size of the scrapie-affected population. Note that in (2.4) each observed scapie affected holding is weighted by the inverse of observing an affected holding of type *i*. The formula (2.4) builds heavily on the Poisson model. To develop an approach robust to violations from the Poisson model an extension of the Zelterman estimate of population size was suggested for covariates [10]. The original suggestion [17] focused on estimating the Poisson parameter by ignoring all counts larger than 2 (which is then evidently robust to contaminations of the data if they only occur in counts larger than 2). The extension (of the Zelterman approach for population size estimation) to include covariates is given as

(2.5)

where the estimates ,  in the linear predicto*r * can be found by means of a logistic regression of the binary variate *B *(*B *= 1 if the scrapie count = 2, *B *= 0 if the scrapie count = 1) on the covariates (here only *x*) in the model. Either estimator, Poisson (2.4) and the generalized Zelterman (2.5), allow for the different forms of the linear predictor *λ *as explained in section 2.2.2. We have run these estimators on SND year-specific data and CSFS data. Note that for the latter, the index cases, those that triggered the incorporation of the scrapie-affected holdings into the CSFS, had to be retained in the dataset.

#### Software

All computational analysis has been done using MINITAB version 15 and STATA version 10.0.

## Results

**Figure 1:**
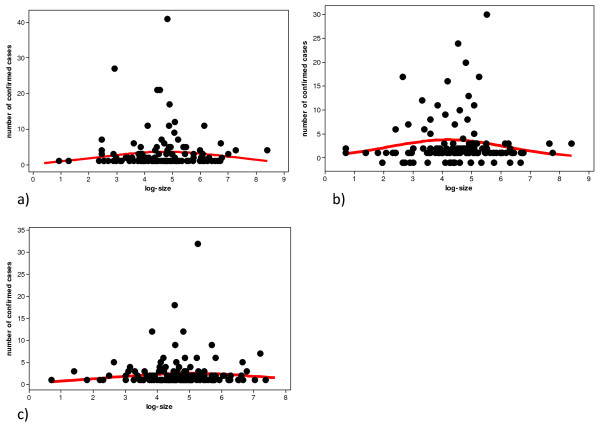
**Scatterplot of the number of confirmed cases of scrapie and log-size of holding in Great Britain for the year 2002 (a), 2003 (b), and 2004 (c) based upon the SND data (solid line is quadratic regression model)**.

First we concentrate on the relationship of the observed scrapie count to the size of the holding. This was possible since for most holdings with positive cases count of scrapie the size of the holding was also available (Table 1). Figure 1 shows this relationship for the three years of SND data separately. There appears to be a curved relationship (increasing trend, then after reaching a maximum, decreasing again) for each year. Note that the size of holding is not constant but experiences large variation (minimum 1, maximum 4433 – see Table 1). This form of relationship is confirmed in the statistical analysis by fitting model 1, model 2, and model 3 to each of the three years 2002, 2003 and 2004 as provided in Table 2. There is clear and strong evidence against model 1. Recall that this model stated the proportionality hypothesis. All model performance measures such as the likelihood, the AIC and BIC never select this model. In fact, there is a large gap between model 1 and model 2 which represents the hypothesis that there is a linear relationship between size of holding and scrapie count. If we compare model 2 with model 3 there is evidence for model 3 consistently over all 3 years. Recall that model 3 represents the hypothesis of curved relationship. The evidence is less strong when model 3 is compared with model 2 (in respect to the comparison of model 2 with model 1). However, note that model 1 is nested in model 2 which is nested in model 3. Hence, model comparisons are also possible on the basis of the likelihood ratio test which establishes that the comparison between model 2 and 3 are significant for all 3 years with a borderline result for the year 2004. These results are important, since they have implications when using the generalized Zelterman regression approach as discussed in (2.5) for estimating the size of the scrapie affected holding population in Great Britain (observed and hidden number of scrapie-affected holdings). Table 2 presents the population estimates from the application of (2.4).

**Table 1:** Demographic characteristics of the SND and CSFS databases: two variables are given – the non-zero case count of scrapie cases per holding and the size of holding

Variable	mean	median	minimum	maximum	n
SND 2002					

*Count of scrapie cases per holding*	3.278	1	1	41	144

*Holding size*	213.2	99	2	4433	125

SND 2003					

*Count of scrapie cases per holding*	3.418	2	1	30	134

*Holding size*	221.7	102.5	1	4433	122

SND 2004					

*Count of scrapie cases per holding*	2.497	1	1	32	151

*Holding size*	192.9	60	2	1577	135

CSFS					

*Count of scrapie cases per holding*	1.614	1	1	18	251

*Holding size*	705.3	502	4	4264	214

**Table 2:** Results of zero-truncated Poisson regression modelling for the offset-model (Model 1: offset is log-size of holding), for the model treating log-size as a free covariate (Model 2) and the model with a quadratic term included (Model 3) for the years 2002, 2003, and 2004 based upon the SND data.  is the scrapie-affected population estimated by means of the generalized Horvitz-Thompson estimator and n the number of holdings confirmed with scrapie by the SND in each year.

	*α* (S.E., ^1^Z)	*β*_1_	*β*_2_		log-likelihood (AIC, BIC)
2002				(n = 144)	

Model 1	-4.3160 (0.0554, -77.92)	1 (fixed)	-	327	-686.43 (1374.9, 1377.7)

Model 2	0.8721 (0.1857, 4.70)	0.0560 (0.0386, 3.43)	-	151	-431.81 (867.6, 873.3)

Model 3	-0.9608 (0.6344, -1.51)	0.9498 (0.2768, 3.43)	-0.1002 (0.0298, -3.36)	153	-423.10 (852.2, 860.7)

2003				(n = 134)	

Model 1	-4.3845 (0.0571, -76.78)	1	-	359	-700.69 (1544.3, 1547.1)

Model 2	1.2485 (0.1889, 6.61)	-0.0239 (0.0405, -0.59)	-	139	-399.90 (803.8, 809.4)

Model 3	-0.8759 (0.6186, -1.42)	1.0482 (0.2841, 3.69)	-0.1252 (0.0324, -3.86)	147	-388.96 (783.9, 792.3)

2004				(n = 151)	

Model 1	-4.5671 (0.0653, -69.98)	1 (fixed)	-	363	-439.16 (880.3, 883.2)

Model 2	0.5373 (0.2810, 1.91)	0.0566 (0.0573, 0.99)		170	-316.82 (637.6, 643.4)

Model 3	-1.0624 (0.9407, -1.13)	0.7776 (0.3953, 1.97)	-0.0773 (0.0412, -1.88)	172	-314.68 (635.3, 644.1)

^1^Z = coeff./S.E.

Table 2 presents the findings from the application of (2.5) on year-specific SND data: evidently the (wrong) model 1 leads to a large overestimation of the number of scrapie-affected holdings whereas estimates for model 2 and 3 appear less different. Model 3 also appears to provide the better choice, in particular, for the year 2003 where population size estimates differ more substantially which is also supported by a significant likelihood ratio test between these two models (LRT = 4.28 P-value = 0.0386). Note the difference in the population size estimates between Tables 1 and 2,  and  respectively. For the best model in each table, Model 3, and any given year, 2003 for example, the generalized Horvitz-Thompson estimator returned a population of 147 holdings, only 13 holdings more than those observed. The generalized Zelterman estimator returned a population of 383 (Table 3).

**Table 3:** Results of generalized Zelterman regression modelling estimating population size based upon the offset-model (Model 1: offset if log-size of holding), the model treating log-size as a free covariate (Model 2) and the model with a quadratic term included (Model 3 for SND year-specific data.

**2002**		CI	log-likelihood
Model 1	705	0 – 3977	-61.70

Model 2	311	199 – 422	-53.07

Model 3	332	166 – 498	-52.62

**2003**			log-likelihood

Model 1	576	0 – 2547	-76.59

Model 2	233	161 – 304	-58.19

Model 3	383	0 – 1037	-56.05

**2004**			log-likelihood

Model 1	672	0 – 3688	-72.07

Model 2	303	206 – 400	-63.95

Model 3	306	201 – 412	-63.83

Table 4 presents the results for the CSFS data. Note that index cases were included in this analysis. Again, we find evidence for the incorporation of a curvature term leading to population size estimates with similar observed-to-hidden scrapie ratios. Moreover, table 4 shows the population estimates from the application of (2.4) and (2.5) to the CSFS data (fourth column). Although these values have no application they are shown for illustrative purposes. As observed in the case of the SND data, (2.4) underestimates the size of the scrapie-affected holding population compared to (2.5). This is likely due to the excessive heterogeneity in the data not captured adequately by the simpler model.

**Table 4:** Results of zero-truncated Poisson regression modelling including population size estimates (columns 4 contains the robust Zelterman estimate in brackets) for the offset-model (Model 1: offset if log-size of holding), for the model treating log-size as a free covariate (Model 2) and the model with a quadratic term included (Model 3) based upon the CSFS data (n = 214).

**Model 1**	coefficient	S.E./Z = coeff./S.E.	(, 95% CI)	log-likelihood (AIC, BIC)
*α*	-6.5573	0.0785/-83.48	457 (1077, 0 – 12,882)	-274.38

*β*_1_	1 (fixed)			(550.76, 554.13)

				

**Model 2**	coefficient	S.E./Z = coeff./S.E.	(, 95% CI)	log-likelihood (AIC, BIC)

*α*	-3.8869	0.6123/-6.35	197 (398, 199 – 597)	-265.92

*β*_1_	0.6164	0.0893/6.90		(535.83, 542.57)

				

**Model 3**	coefficient	S.E./Z = coeff./S.E.	(, 95% CI)	log-likelihood (AIC, BIC)

*α*	1.6045	1.0116/1.59	228 (403, 200 – 606)	-257.76

*β*_1_	-1.2240	0.3430/-3.57		(521.52, 531.61)

*β*_2_	0.1486	0.0291/5.11		

Table 5 shows the relationship of holding size to the number of cases in CSFS holdings *after the index case was removed from the case count*. Hence, we have now zero counts in the data set, in fact, a lot more than can be captured with the conventional Poisson regression model. For these instances the *zero-inflated *Poisson regression model (2.3) has been developed and used in the analysis. The results in Table 5 show again the various statistics required to evaluate the models 1, 2 and 3. We find here again that the simple proportionality model is unsuitable. Both selection criteria, AIC and BIC, reject the model 1 and choose model 3 as the more appropriate model. Also the values of the likelihood ratio tests, namely for the comparison of model 1 and model 2 with LRT = 2 [-232.63-(-236.27)] = 7.28 and for the comparison of model 2 and model 3 with LRT = 2 [-223.99-(-236.27)] = 17.28, lead to prefer model 3. Hence, also from this analysis we find evidence for a violation of the hypothesis of proportionality.

**Table 5:** Results of zero-inflation Poisson regression modelling for the offset-model (Model 1: offset if log-size of holding), for the model treating log-size as a free covariate (Model 2) and the model with a quadratic term included (Model 3) based upon the CSFS data without index cases in the case count (n = 214).

**Model 1**	coefficient	S.E.	Z = coeff./S.E.	log-likelihood (AIC, BIC)
*α*	-6.0402	0.0981	-61.55	-236.27
	
*β*_1_	1 (fixed)			(476.54, 483.27)

				

**Model 2**	coefficient	S.E.	Z = coeff./S.E.	log-likelihood (AIC, BIC)

*α*	-4.0355	0.2789	-5.54	-232.63
	
*β*_1_	0.7140	0.1043	6.84	(471.27, 481.37)

				

**Model 3**	coefficient	S.E.	Z = coeff./S.E.	log-likelihood (AIC, BIC)

*α*	2.1013	1.1687	1.80	-223.99
	
*β*_1_	-1.3656	0.0333	5.07	(455.99. 469.45)
	
*β*_2_	0.1686	0.0270	-1.24	

**Figure 2:**
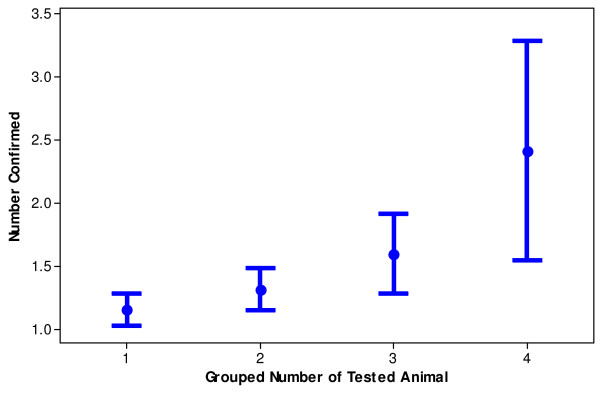
**Interval plot (mean with 95% CI) of number of confirmed cases of scrapie against the grouped number of tested animals (groups were determined on the basis of the quartiles) for the CSFS data**.

Finally, we consider instead of the size of holding the *number of tested animals *as a covariate in the analysis. This covariate is available in the CSFS data and can be utilized for this analysis. The results are presented in Table 6. Note that here is clear support for the model 1 (proportionality) based on the AIC and BIC. Also, note that the LRT = 0.08 which is not significant with 1 df. Hence, it can be concluded that for *this type of covariate *we find clear evidence for the presence of proportionality. This proportionality is also illustrated in Figure 2.

**Table 6:** Results of zero-inflation Poisson regression modelling for the offset-model (Model 1: offset if log-size of number of tested animals), for the model treating log-size as a free covariate (Model 2) and the model with a quadratic term included (Model 3) based upon the CSFS data with index cases removed from the data set (n = 174).

**Model 1**	coefficient	S.E.	Z = coeff./S.E.	log-likelihood (AIC, BIC)
*α*	-4.3154	0.1091	-39.56	-208.00
	
*β*_1_	1 (fixed)			(420.00, 426.32)

				

**Model 2**	coefficient	S.E.	Z = coeff./S.E.	log-likelihood (AIC, BIC)

*α*	-3.9731	1.1692	-3.40	-207.96
	
*β*_1_	0.9332	0.2275	4.10	(421.92, 431.40)

				

**Model 3**	coefficient	S.E.	Z = coeff./S.E.	log-likelihood (AIC, BIC)

*α*	-6.3097	5.1816	-1.22	-207.83
	
*β*_1_	1.9721	2.2259	0.89	(423.67, 436.30)
	
*β*_2_	-0.1130	0.2387	-0.47	

## Discussion

Our results show that the relationship between the holding size and the number of confirmed scrapie cases followed a curved line for the years 2002, 2003 and 2004 of SND data. The scrapie count increases with holding size with a peak around holding sizes of 100 adult sheep after which the scrapie case count decreases again. This pattern not only appears consistently for the SND in the 3 years, it also occurs in the same way for the CSFS data set. This similarity between these two sets of analysis was expected due to the large weight of the SND data (70.6%) in the CSFS dataset.

In broad terms, the observed curved relationship between the case count and the holding size can be a manifestation of the dynamics of infection within the holding or an artefact related to the reporting and/or testing of cases; or more likely, a combination of both. We can only speculate about the processes potentially responsible for the observed relationship. Under a biological approach, only the exhaustion of all susceptible animals would stop the progression of the epidemic within holding. For scrapie, where the outcome is always fatal, the exhaustion of the susceptible population is only achieved by means of depopulation or selection of resistant genotypes to the disease [18]. To relate either intervention to our results, their effects would have to be different depending on the holding size. Larger holdings might have pursued genotyping of their animals at a greater rate than smaller ones. Or, similarly, the increased number of movements into larger holdings might have facilitated the shift in their genotype profiles towards resistant types more rapidly than in smaller, more static holdings. Either option might explain the reduced number of scrapie cases observed at the right end of our data.

The effect of reporting artefacts appears plausible and allows meaningful interpretation of our SND results. We could hypothesize a proportional relationship between the holding size and the level of supervision, defined in this setting as the regular observation of the holding by the farmer. It follows that with greater supervision, all other things equal (e.g. no biological effects associated with the occurrence of disease as suggested above), we should expect, from a larger holding, a greater case count. Our results on SND data show different indicating that some of the steps in the logic above do not hold. To test the rationale above, for SND data, is not straightforward as there is no clear definition of "supervision" and even less clear approach to its quantification and measurement. Our analyses of the CSFS data could provide some proxy indication of the relationship between supervision and case count. Within the CSFS, the TSE-testing equates to the supervision in SND holdings. More specifically, our results show the proportional relationship between the number of samples tested within holding and the number of cases detected in the CSFS. This is different from the curved pattern observed with the SND data and would indicate the lack of proportionality between holding size and supervision in the field. This explanation would fit well with the large body of evidence that support the occurrence of reporting artefacts affecting the ascertainment of scrapie [3,4,9,10,12,19]. It would appear intuitive to think that larger holdings may have more difficulty in identifying all the cases of scrapie. This reduced detection capability would account for the significant quadratic relationship in the case of the SND datasets.

It appears difficult to reconcile our results with those of previous works [2,3,20] where larger holdings appeared at greater risk of having scrapie. On the other hand, it was shown that the risk of scrapie followed a quadratic shape with increasing holding size for Great Britain and SND data from 1993 to 2002 [6]. Note that this previous work compares counts of holdings stratified by holding size with the occurrence of scrapie regardless of the number of cases within holding. The results presented here are not directly comparable as we focus on the count of confirmed scrapie cases within holding and hence, we are not informing any measure of risk of scrapie. However, our present results and those of [6] are consistent with the occurrence of reporting artefacts: a non-proportional supervision in larger holdings will result in fewer of them reporting suspect cases.

Our results on the zero-inflated CSFS dataset inform a pre-clinical stage and, by definition, one free from human-related artefacts. They also refer to a different disease stage, infection, from that, clinical disease, shown by the analyses on SND data. Unfortunately, two sources of biases are still pertinent to our CSFS results. The first one originates from the sampling scheme performed within the CSFS, as per EU requirements, by which sampling stops at 150 animals regardless of the holding size [8]. This sampling strategy is aimed at detecting, with an assumed within-holding prevalence of 2%, at least one infected animal. Sampling for detection of disease does not favour case counts in larger holdings [21]. The second source of biases, a less certain one, would affect the representativeness of our results on the general scrapie-affected population. In [8] a large proportion of CSFS holdings with just one confirmed case of scrapie is reported, the index case. This might reflect the active search of scrapie cases by farmers in order to benefit from the compensation schemes introduced with the CSFS in 2004. Indeed, a significant increase in the number of cases in some areas in Great Britain around the introduction of the CSFS was reported recently [22].

### Scrapie-affected population

It would seem natural to expect that the case count rose linearly if not proportional with the holding size and, had this been correct, it could be used beneficially in estimating the hidden burden of the scrapie epidemic. The offset-model (proportionality hypothesis) was considered in [11] and needs to be revised on the basis of these findings here. For example, for SND data and 2002 and 2004, the proportionality model returns estimates of the scrapie-affected population twice as large as those returned by the model with the best fit, that with the quadratic term. Clearly, the appropriate choice of a model is crucial for deriving an appropriate estimate of the population size of scrapie affected holdings. It appears founded then that for the surveillance schemes of SND and CSFS the hypothesis of proportionality does *not *hold. It was also shown that the robust generalised Zelterman estimation based upon fitting a logistic model [10] with quadratic terms for the year-specific SND data will lead to a more realistic value of the population size. Note that the appropriate choice of the model is not only crucial for avoiding a spurious population size estimate of scrapie (see Table 2), it is also important for achieving valid variance estimates leading to trustworthy confidence intervals. As Table 3 shows, all confidence intervals computed on the basis of the – inappropriate – model 1 are meaningless since they are too large. For the robust regression model the inclusion of the quadratic term is less consequential: for the years 2002 and 2004 the population size estimates based upon model 2 and model 3 are quite close (see Table 3) whereas for the year 2003 model 2 will be preferred. We can conclude from this analysis that it is less important to consider quadratic terms in the robust generalized Zelterman approach. This can easily be explained since the robust approach ignores higher scrapie counts in the holdings and, hence, is less sensitive to model changes affecting only larger scrapie counts.

Our efforts to prevent the effect of reporting artefacts on the relationship between holding size and the case count concentrated on the analyses of CSFS data after the removal of the index case. This is also a more realistic scenario as the accumulation of clinical cases within holding is no longer possible after the introduction of the CSFS in 2004 [12]. For the latter dataset, and to increase the number of observations (holdings) in our lists, we joined the two years of data, 2005 and 2006. The joint list prevented the estimation of a biologically meaningful measure of frequency from this dataset and, hence, comparisons with previous works [12].

### Practical rationale

In general terms, our results have two applications: 1) for the correct adjustment of population estimates of scrapie-affected holdings and 2) to advice on the correct relationship between holding size and the count of scrapie cases within holding. The former constitutes one of the first and most basic parameters in surveillance planning. The latter will help in the development of predictive models and, on a more practical side, in the refinement and increased understanding of targeted surveillance approaches to classical scrapie. It is important to stress at this point that our results are applicable only to the classical form of scrapie. Clinical atypical scrapie was first diagnosed in Great Britain in 2005 [23], after the introduction of the CSFS and, so far, only one holding has shown multiple cases of atypical scrapie (Del Rio Vilas, personal communication). It is worth mentioning that the testing regime applied under the CSFS scheme allows the discrimination of the two types of scrapie [8].

The implications of our results are large, if not for what they are, for what they show: either a biased, for the SND, or a truncated, for the CSFS, picture of the effects of holding size in the case count within holding. This limitation affects our understanding of the results derived from the scrapie surveillance. The current active surveillance for scrapie is a targeted one: i) at the individual level by targeting sheep older than 18 months of age where the likelihood of detecting infection is greater and ii) at the holding level by, inadvertently, targeting large holdings [24]. The latter requires careful consideration after our results. The occurrence of under-ascertainment in larger holdings by means of reduced supervision will affect the detection capability of one of the surveys, the fallen stock. The lack of opportunity to spot disease can easily be extrapolated to insufficient supervision to identify and promptly report the dead-on-farm sheep. This might contribute to explain the reduced average size of scrapie-affected holdings detected by this survey relative to those detected by the abattoir survey [24].

Reporting artefacts should not be a problem for the abattoir survey due to its random nature. Farmers can do little to influence the sampling approach of the survey. Only a biological effect, e.g. a greater proportion of resistant genotypes in larger holdings, might affect the results from this survey. In fact, if there was such a biological effect, the natural tendency of the survey to over-sample larger holdings might result in the underestimation of the prevalence of scrapie. This effect might also explain the reduced sensitivity of the abattoir survey relative to the fallen stock [25].

## Conclusion

With the very low numbers of detected scrapie in recent years [1] there appears to be little rationale to support a consistent sampling scheme that focuses on efforts to identify all the scrapie cases within the CSFS holdings. The very detection of scrapie cases implies the end of their infectiousness as they are detected, either through fallen stock or regular culls, at the point of removal from the holding. It is those not detected through the truncated sampling regimes that pose a risk. Our results do not allow knowing if the reduced number of scrapie cases in CSFS holdings is the result of the truncated sampling regime or there is no such effect and large holdings, for whatever reason, present fewer cases of scrapie. Only the full TSE-testing of a representative sample of CSFS holdings would answer this question. The lessons from that exercise would allow in-depth assessment of the power of the current control measures, and in particular, of the sampling scheme to detect and eliminate all cases of scrapie from the holding.

## Competing interests

The authors declare that they have no competing interests.

## Authors' contributions

VDRV wrote the background, discussion and conclusion section. DB wrote the methods and results section and contributed the statistical analysis. Both authors read and revised the entire paper jointly.
